# Supplementary material for: Theranostic Properties of Crystalline Aluminum Phthalocyanine Nanoparticles as a Photosensitizer
Source: Pharmaceutics. 2022 Oct 6;14(10):2122. doi: 10.3390/pharmaceutics14102122 (PMC9611939; doi:10.3390/pharmaceutics14102122)
Supplement: Supplementary file 1 [file pharmaceutics-14-02122-s001.zip › pharmaceutics-1927616-supplementary.pdf]

Supplementary Materials: Theranostic Properties of Crystalline Aluminum Phthalocyanine Nanoparticles as a Photosensitizer

Vladimir I. Makarov, Daria V. Pominova, Anastasiya V. Ryabova, Igor D. Romanishkin, Arina V. Voitova, Rudolf W. Steiner and Victor B. Loschenov

Experimental setups and methods for Estimation of Fluorescence Quantum Yield and Photodynamic Efficiency of aluminum phthalocyanine nanoparticles are shown in Figure S1.

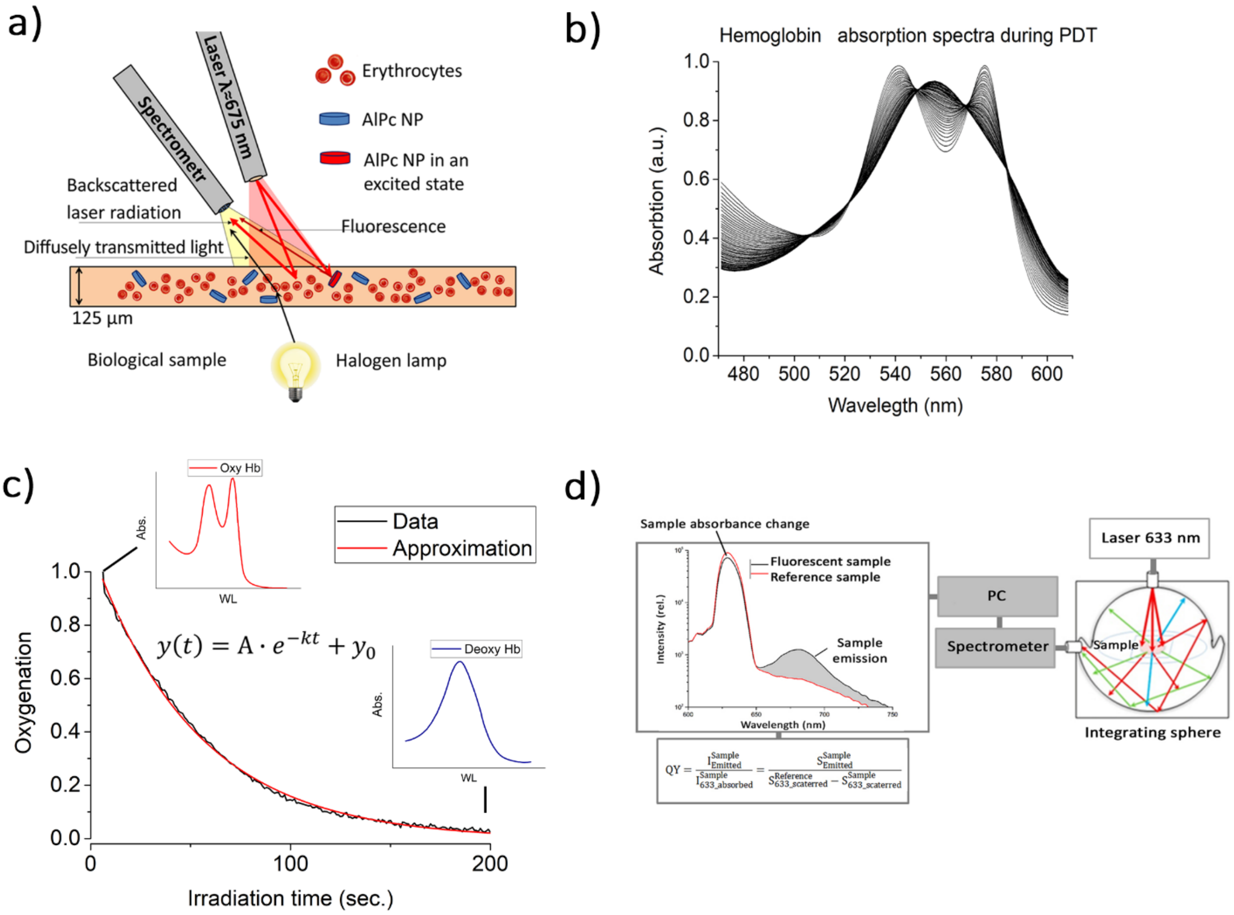

Figure S1. (**a**) Measurement scheme for evaluating the photodynamic efficiency of PS; (**b**) absorption spectra of hemoglobin during the transition from oxy- to deoxygenated form [39]; (**c**) the dependence of hemoglobin oxygenation in the sample on the duration of laser exposure and the parameters of its approximation by an exponential function; (**d**) scheme of the experimental setup for determining the quantum yield of PS fluorescence in a sample [35].

Figure S2 (top) shows the x-ray diffraction pattern measured from AlPc microparticles powder. The particles are molecular crystals of the triclinic syngony of the P1 space group with the lattice parameters shown in Figure S2 (bottom).

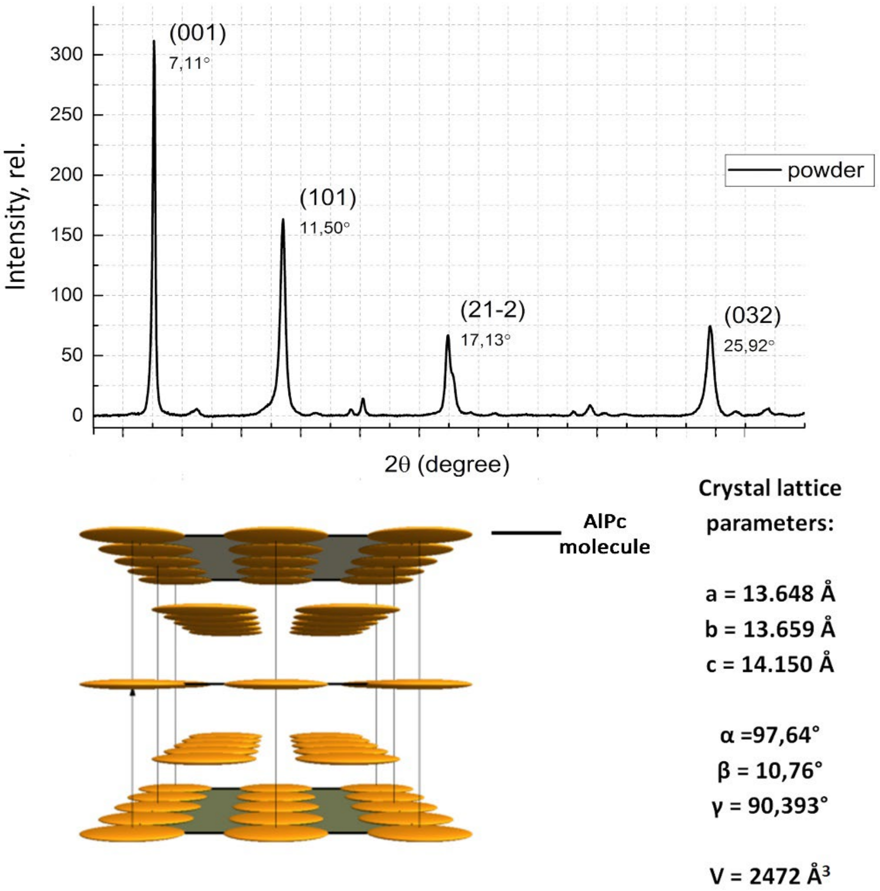

Figure S2. XRD spectrum measured from AlPc microparticles powder. The particles are molecular crystals of the triclinic syngony of the P1 space group with the lattice parameters as shown.

Figure S3 shows XRD spectra measured from AlPc microcrystal powder and from AlPc NPs colloids in various media (saline, blood serum, RPMI cell medium, phosphate-buffered saline PBS) after 3 h of incubation.

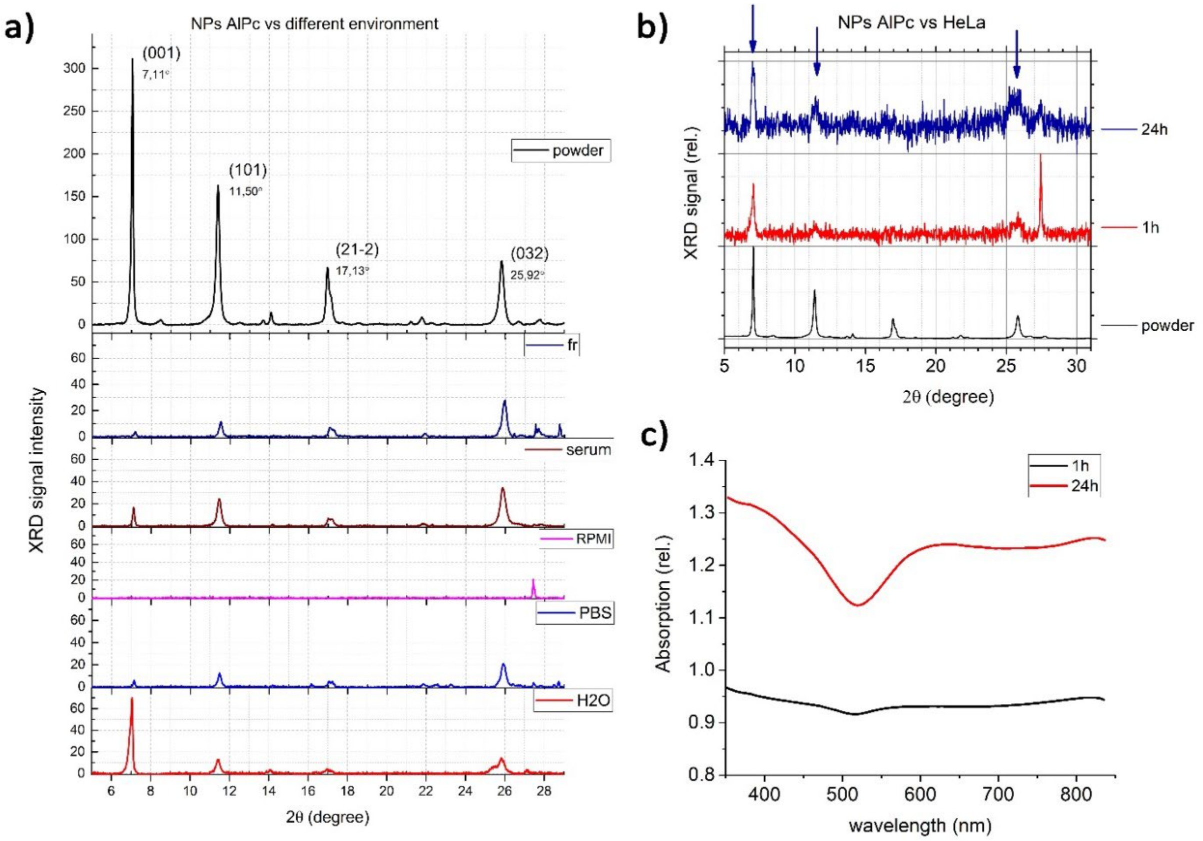

Figure S3. (**a**) XRD spectra of AlPc microparticles (powder) and AlPc NPs in saline, blood serum, RPMI nutrient medium, phosphate-buffered saline (PBS) and water; (**b**) XRD spectra of HeLa cells 1 and 24 h after incubation with AlPc NPs; (**c**) absorption spectra of AlPc NPs after incubation with HeLa for 1 and 24 h.

The broadening of the diffraction peaks with time indicates a decrease in the average size of AlPc NPs inside the cells. Figure S4 shows the scattered laser radiation and fluorescence spectra of HeLa cells with AlPc photosensitizer in molecular and nanoform.

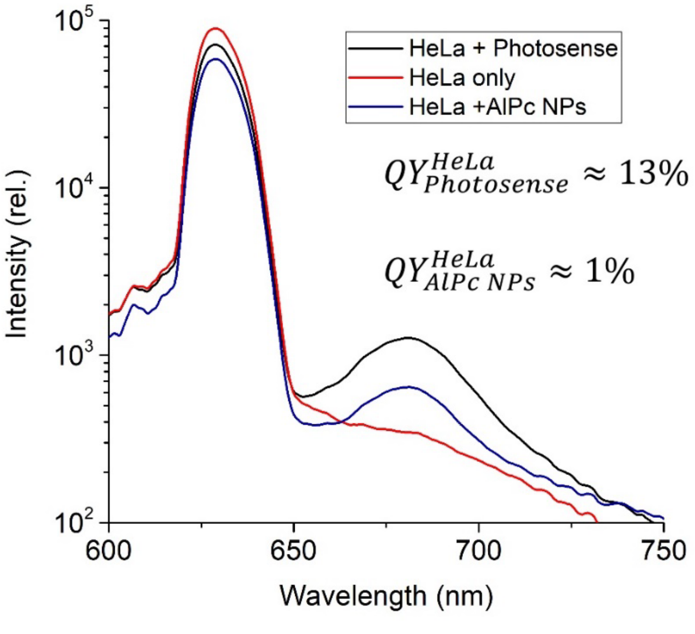

Figure S4. Spectra of scattered laser radiation (left peak) and fluorescence (right peak) of HeLa cells with the AlPc photosensitizer in molecular and nanoform, as well as calculated values of the fluorescence quantum yield.
